# Obstructive sleep apnea: is there a difference between vertical and horizontal laryngectomy?

**DOI:** 10.5935/1808-8694.20130124

**Published:** 2015-10-08

**Authors:** Raquel Chartuni Pereira Teixeira, Michel Burihan Cahali

**Affiliations:** a M.D. (MSc. Student, Public Servant State Hospital of São Paulo); b Ph.D. (Assisting Physician, Public Servant State Hospital of São Paulo)

**Keywords:** laryngectomy, obstructive, sleep apnea, spirometry

## Abstract

Partial laryngectomy is used in the treatment of laryngeal cancer. Structural alterations of the upper airway arising from partial laryngectomy can cause obstructive sleep apnea (OSA).

**Objective:**

To compare the prevalence and severity of OSA in patients submitted to horizontal and vertical partial laryngectomy and assess the role of spirometry for these patients.

**Method:**

Cross-sectional clinical study with individuals offered partial laryngectomy. The included patients were assessed through interview, upper airway endoscopy, polysomnography, and spirometry.

**Results:**

Fourteen patients were evaluated and 92.3% were found to have OSA. The apnea-hypopnea index was significantly higher among patients submitted to vertical laryngectomy (mean = 36.9) when compared to subjects offered horizontal laryngectomy (mean = 11.2). The mean minimum oxyhemoglobin saturation was 85.9 in the horizontal laryngectomy group and 84.3 in the vertical laryngectomy group. Spirometry identified extrathoracic upper airway obstruction in all patients with OSA.

**Conclusion:**

The studied population had a high incidence of obstructive sleep apnea. OSA was more severe in patients offered vertical laryngectomy than in the individuals submitted to horizontal laryngectomy. Spirometry seems to be useful in the detection of cases of suspected OSA, as it suggests the presence of extrathoracic upper airway obstruction.

## INTRODUCTION

Partial laryngectomy has been used in cases of non-advanced laryngeal cancer to preserve some of the larynx's vital functions, by sparing the natural airways, protecting the lower airways, and preserving the patients' voice. Structural alterations to the upper airways consequent to partial laryngectomy may predispose individuals to obstructive sleep apnea (OSA), a condition seen in 81% to 91% of these patients^1^.

Two approaches are possible in partial laryngectomy: horizontal partial laryngectomy (HPL) and vertical partial laryngectomy (VPL). In HPL, as in supraglottic laryngectomy, the entire supraglottis - including the epiglottis, the vestibular folds, Morgagni's ventricles, and the hyoid bone - is removed. In supracricoid laryngectomy - a horizontal procedure - the entire supraglottis is removed, along with the vocal folds, the pre-epiglottic and paraglottic areas, and the thyroid cartilage. At least one arytenoid must be spared to preserve phonation and sphincteric function^2^. The cricoid is spared and sutured to the hyoid. The neoglottis is then relocated to the cricoid. In these cases, the area of the cricoid cartilage and remaining arytenoid is elevated and fixated against the epiglottis, in a reconstruction procedure called cricohyoidoepiglottopexy (CHEP). When the epiglottis is removed, the reconstruction is carried out by suturing the cricoid and arytenoids to the hyoid, in a procedure called cricohyoidopexy (CHP). In VPL, the vocal folds, the laryngeal ventricle, the vestibular fold, and the lamina of the thyroid cartilage on the side of the lesion are resected, sparing at least one portion of the thyroid cartilage to preserve laryngeal support. Part of the contralateral vocal fold may also be removed and one of the arytenoids could be completely or partially resected. The external perichondrium of the resected thyroid cartilage is used in the reconstruction of the affected area, to produce a new laryngeal wall^3^.

A study reported similar incidences and severity of OSA secondary to HPL or VPL, although the opening of the neoglottis is larger in patients submitted to VPL^1^.

Spirometry is used to assess lung function4., 5.. The spirometry flow-volume curves of patients with OSA usually fail to show a pattern of upper airway extrathoracic obstruction^6^; and obstructive lung disease is not an independent risk factor for OSA^7^. However, this test can detect functional anomalies when the diameter of the pharynx or larynx has been reduced to 8 mm or less8., 9., 10.. In these cases, the expiratory flow-volume curve may seem normal, but the inspiratory portion of the curve is typically flat, suggesting high extrathoracic obstruction.

Since the size of the smaller area of the larynx seems to be correlated with OSA in patients offered partial laryngectomy^1^, it is possible that high extrathoracic obstruction verified by spirometry could be used to identify individuals at risk of developing OSA. The prevalence of OSA in patients submitted to laryngectomy was compared to the prevalence seen in the general population. This study also aimed to compare the presence and severity of OSA between groups of patients submitted to vertical or horizontal partial laryngectomy and to assess the role of spirometry in detecting OSA in laryngectomy patients.

## METHOD

This study was approved by the Research Ethics Committee of the institution and given permit 001/10. The individuals enrolled in this study signed informed consent terms.

Patient charts were reviewed to select the individuals submitted to partial laryngectomy at the ENT service of our institution between 2000 and 2008. The studied population included patients currently aged 18 and older submitted to one partial laryngectomy procedure to treat laryngeal cancer with removal of part of the laryngeal framework and maintenance of their natural airways at the time of the study; subjects offered temporary tracheostomy who had their natural airways at the time of the study; and individuals offered other approaches to larynx cancer such as neck dissection, radiation therapy, and/or chemotherapy.

Patients with tracheostomies, even if occluded, individuals submitted exclusively to cordectomy, subjects with local or regional relapsing disease or tumor metastasis, patients on drugs that alter neuromuscular control, individuals with congestive heart failure (CHF) or lung diseases requiring oxygen therapy were excluded. Six of the 40 selected patients were excluded for having relapsing disease; three died of non-tumor related causes; four had chronic obstructive pulmonary disease requiring oxygen therapy; and two had CHF. Three individuals did not agree to join the study. One was excluded because the tracheostomy could not be removed after surgery. Seven patients could not be contacted. Therefore, 14 patients were enrolled in the study.

Choice of treatment:

Seven patients underwent PHL and seven had PVL.

In the PHL group, four individuals had T2 tumors and three had T1 tumors at the time of diagnosis. Six were treated with supracricoid laryngectomy. Supraglottic laryngectomy was offered to one patient. In this group, three patients had postoperative radiation therapy; three had elective bilateral neck dissection and four had unilateral neck dissection. None of the patients underwent postoperative chemotherapy. Four of the six patients submitted to supracricoid laryngectomy had reconstruction surgery by CHP and two by CHEP.

In the PVL group, four patients were diagnosed with T2 tumors and three with T1 tumors. Four subjects underwent fronto-lateral laryngectomy and three to hemilaryngectomy. Two individuals had postoperative radiation therapy. Three had elective unilateral neck dissection. None of the patients had postoperative chemotherapy. Three patients required airway reconstruction with platysma myocutaneous flaps.

Patients were followed up for a mean time of 54 months from the end of treatment to polysomnographic evaluation (6 to 84 months).

### Clinical assessment

Patients were analyzed based on the type of surgery (PHL or PVL) they underwent, complaints of snoring, the temporal correlation between snoring and surgery (if the patients snored before or after surgery; if snoring worsened after surgery), and measurements of neck circumference and body mass index (kg/m^2^). Diurnal sleepiness was assessed through the Epworth scale, and excessive sleepiness was attributed to individuals with scores greater than 10. Relapsing tumors and the size of the laryngeal airways of all patients were assessed using a fiberoptic endoscope. None of the patients had signs of relapsing disease or complaints of dyspnea. The size of laryngeal airways was deemed safe for the maintenance of natural airway in all cases.

### Polysomnography

Enrolled patients were sent to a sleep lab to undergo nocturnal polysomnography. The following parameters were monitored: electroencephalogram, electro-oculogram, electrocardiogram, chin and lower limb electromyograms, oral and nasal respiratory flow with a cannula and thermistor, respiratory effort wearing chest and abdominal belts, oxygen saturation on a digital oximeter, snore sensor, and position sensor. The tests were staged based on the 2005 AASM criteria^11^, particularly the recommendations on how to detect hypopnea.

These tests were used to assess the apnea hypopnea index (AHI) and baseline, minimum and maximum oxyhemoglobin saturation.

### Spirometry

Patients were submitted to spirometry to assess the presence of high extrathoracic obstruction. They were asked to perform a forced inspiratory maneuver at the end of expiration to check whether they would produce the typical flattened flow-volume curve seen in high extrathoracic obstruction^4^. The data for at least three acceptable maneuvers was captured for each patient to verify test reproducibility. The maneuvers were deemed acceptable when the peak expiratory flow was 10% or 500 ml greater than the peaks attained in previous maneuvers.

### Statistical analysis

Given the small number of enrolled patients and the normal distribution pattern followed by most of the analyzed variables, non-parametric statistical tests were used to compare the two procedures. In this statistical analysis approach, median values and their respective interquartile ranges were used as central tendency measurements. In order to compare this and other studies, some of the results were also presented in the form of mean values. The comparisons between both groups were made using the Mann-Whitney U test. The impact of other variables upon the differences in AHI was shown through Spearman's rank correlation coefficient. Statistical significance was assigned to events with a *p*-value equal to or lower than 0.05. Statistically significant differences in the tables were signaled with an asterisk (^*^).

## RESULTS

Thirteen males and one female were enrolled in the study. Median age at the time of assessment was 67.5 years (interquartile 11 years), and mean age was 64.9 years (41–84 years). Median BMI was 27.3 kg/m^2^ (interquartile 5.4 kg/m^2^), and mean BMI was 25.7 kg/m^2^ (19.4–29.4 kg/m^2^). Median neck circumference was 38.5 cm, ranging from 32 to 49 cm, and mean neck circumference was 39.6 cm.

The female patient and 12 male subjects (92.9%) snored at night while sleeping, and nine reported to have started snoring or worsened their snoring significantly after partial laryngectomy. The tests were carried out a mean of 67.2 months after surgery (9-102 months). Excessive sleepiness (Epworth > 10) was observed in four patients (28%).

The mean AHI was 24.0 (3.9–72.3). According to polysomnography findings, 13 patients (92.9%) had OSA (AHI > 5). Mild OSA (5 < AHI < 30) was seen in 42.9% of the patients; 21.4% had severe OSA (IAH > 30). A significant correlation was found between AHI and BMI (s = 0.76, *p* = 0.002), neck circumference (s = 0.61, *p* = 0.02), and scores in the Epworth sleepiness scale (s = 0.77, *p* = 0.001). Mean baseline saturation, minimum and mean oxyhemoglobin saturation in the tests were 94%, 89% and 93%, respectively.

Endoscopic examination of 12 patients revealed edema in the interarytenoid area and reduced larynx lumen. The differences between the five patients in the PHL group and the seven in the PVL group were not statistically significant. In the PVL group, prolapse of the laryngeal framework in the operated side into the larynx lumen was seen in five patients during forced inhalation.

Spirometry showed high extrathoracic obstruction in 13 of the 14 patients, seven in the PVL and six in the PHL group. The patient with normal spirometry results had an AHI < 5 (3.9).

**Graph 1:**
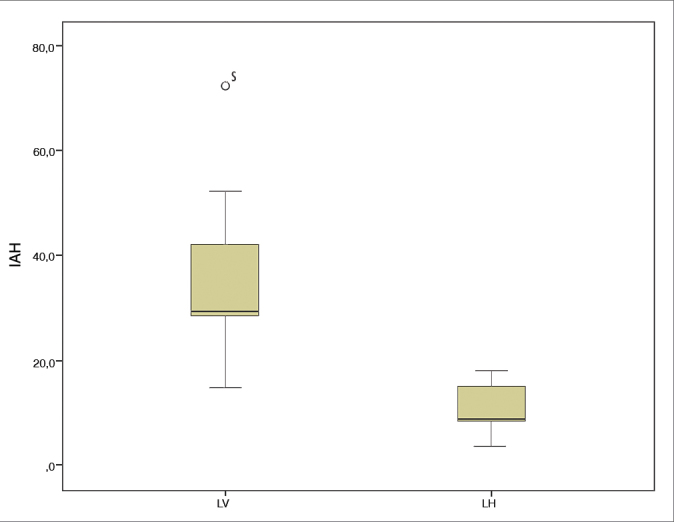
IAH: Apnea hypopnea index; LV: Partial vertical laryngectomy; LH: Partial horizontal laryngectomy.

Anthropometric, clinical, and polysomnogram data for the PHL and PVL groups are shown in Table 1. The AHI was significantly higher in the PVL group (*p* = 0.004), as shown in Graph 1. Additionally, the PVL group had significantly worse minimum oxyhemoglobin saturation levels and significantly higher scores in the Epworth sleepiness scale. Neck circumference was larger, albeit not statistically, in the PVL group. No differences were seen between groups in regards to patient gender and the other analyzed parameters.

**Table 1:** Anthropometric, clinical, and polysomnogram data of the partial vertical laryngectomy (PVL) and partial horizontal laryngectomy (PHL) groups.

Parameter		PHL Group			PVL Group		*P*
	Mean	Median	Range	Mean	Median	Range	
Age (years)	66.6	67.0	55 - 81	63.3	68.0	41 - 72	0.90
BMI (kg/m^2^)	24.3	25.6	19.4 -28.0	25.7	26.7	22.6 - 29.4	0.16
Neck circumference	37.4	39.0	32.0 - 42.0	41.9	43.0	35.0 - 49.0	0.20
AHI	11.2	9.1	3.9 - 18.0	36.9	29.3	15.1 - 72.3	0.004^1^
Minimum O_2_ (%)	85.9	86.0	83 - 89	84.3	85.0	79 - 87	0.01^1^
Epworth	7.1	7.0	5 - 9	10.0	9.0	9 - 14	0.015*

* *p* < 0.05 (Mann-Whitney test).

No statistically significant differences were observed between patients submitted to radiation therapy and individuals not submitted to radiation therapy in terms of presence or severity of OSA. The AHI of the five patients submitted to radiation therapy was greater than five (mean = 25.1). Eighty-eight percent of the nine patients who did not undergo radiation therapy had an AHI greater than five (mean = 27.01; *p* = 0.06).

## DISCUSSION

Although the patients submitted to partial laryngectomy with maintenance of the airways did not report diurnal dyspnea, the respiratory impact of surgery in their sleep is not known. Our study found that 92.9% of the patients submitted to partial laryngectomy had OSA, while the subjects offered PVL had significantly more severe manifestations of OSA than the patients submitted to PHL. Spirometry revealed upper airway extrathoracic obstruction in 92.3% of the patients; only one patient with normal spirometry test results did not have OSA.

An epidemiological study looked into the presence of OSA in the same region and in patients within a similar age range as that of our study, and reported significantly lower prevalence of OSA (34.5%)^12^.

The association between OSA and treatment of head and neck tumors has been discussed by a number of authors. A study^13^ reported a prevalence rate of 91.7% in patients with AHI > 15 in a group of 24 patients treated for various types of head and neck tumors. In our study, the prevalence of AHI greater than 15 was 50%. This difference may be attributed to the enrollment, in the aforementioned study, of patients treated for tumors of the base of the tongue and pharynx; either of these areas is equipped with a hard laryngal framework, which makes them more prone to collapse during sleep.

Why was OSA more severe in the PVL patients when compared to individuals offered PHL? CT scans of the larynx taken in the late postoperative follow-up of partial horizontal laryngectomy patients indicate the size of the cricoid cartilage ring remained unaltered^14^. There is little material of soft tissue density in the lumen of the cricoid ring after PHL^15^. Additionally, the neoglottis is placed in a more cranial position than the normal larynx, given the reduction of the vertical axis of the larynx^13^. The relocation of the larynx toward the hypopharynx possibly reduces the propensity of the airway to developing OSA, as the pharynx is now shorter^16^.

In PVL, there is an increase in the amount of soft tissue in the pre-epiglottic region of the operated side. The larger axis of the glottis ceases to exist in the anteroposterior position and is rotated toward the resected side, potentially narrowing the neoglottis^17^. Although the neoglottis produced after PVL has more air space than the one resulting from PHL, it loses half of its support due to the removal of a wing of the thyroid cartilage. Thus, the propensity to local collapses due to reduced muscle tone during sleep seems to be increased in PVL.

Evidently, our study has its limitations. It is a retrospective study with a small series. Although polysomnography was not carried out before surgery, 64.3% of the patients reported to have started snoring or to have worsened their snoring after surgery, suggesting a likely correlation of cause and effect. A larger sample of subjects with normal spirometry test results - a relatively rare occurrence, given the narrowing of the airways produced by partial laryngectomy - is required to establish the correlation between spirometry and OSA. The age range of our patients and the prevalence of smokers in this population result in significant incidence of death for other causes such as cardiovascular disease. When added to the lack of willingness of some patients and their families of joining the study, this factor limited the number of subjects enrolled in our study.

Matyja et al.^18^ used spirometry to assess the airways of 39 patients submitted to PHL and of 25 offered PVL. The majority of the patients - 85% of the subjects in group PHL and 72% in the PVL group - had flat flow-volume curves suggestive of high extrathoracic obstruction, as also seen in our study. Spirometry may have a role in the screening of patients submitted to partial laryngectomy suspected for OSA, once the only patient without OSA was also the only one to have normal spirometry test results. Spirometry is a simple way to assess the functional impact of having surgically narrowed upper airways.

## CONCLUSION

Higher incidences of OSA were observed in patients submitted to partial laryngectomy when compared to the general population within the same age range. OSA was more severe in patients submitted to partial vertical laryngectomy than in individuals offered partial horizontal laryngectomy. More data is required to assess the value of spirometry in the screening of patients suspected for OSA in this group.
